# A novel mouse model of adenine-supplemented high-fat diet induced cardiovascular-kidney-metabolic syndrome

**DOI:** 10.1038/s41440-026-02645-1

**Published:** 2026-04-27

**Authors:** Hiroe Ono, Yoichiro Otaki, Tetsu Watanabe, Ryuhei Yamaguchi, Tomohiro Takehara, Shingo Tachibana, Jun Goto, Takanori Arimoto, Haruki Ochi, Masafumi Watanabe

**Affiliations:** 1 Department of Cardiology, Pulmonology, and Nephrology, Yamagata University School of Medicine, Yamagata, Japan; 2 Department of Biology, Graduate School of Science and Faculty of Science, Kobe University, Hyogo, Japan

**Keywords:** CKM syndrome, Cardiac hypertrophy, Implemental hypertension, Kidney atrophy

## Abstract

Cardiovascular-kidney-metabolic (CKM) syndrome, a recently proposed concept focusing on the interrelationship among cardiovascular system, chronic kidney disease, and metabolic risk factors, is associated with high morbidity and mortality. The mechanism of CKM syndrome has not yet been fully examined due to the lack of an animal model. Here, we investigated whether an adenine-supplemented high-fat diet (AHFD) can induce CKM syndrome in mice. We fed normal chow diet (NCD), adenine-supplemented diet (AD), high-fat diet (HFD), and 0.15% AHFD to 129×1/Sv mice for 16 weeks and 0.2%AHFD to 129×1/Sv for 6 weeks. Also, C57BL/6 N mice were fed with 0.15%AHFD for 16 weeks. Metabolic parameters, blood pressure, organ weights, histology, and RNA sequencing were analyzed. The 0.15%AHFD group exhibited hypercholesterolemia, elevated blood pressure, kidney atrophy with fibrosis, and cardiac hypertrophy with interstitial fibrosis. Both kidney and cardiac RNA sequencing in the 0.15%AHFD group revealed upregulation of inflammatory and immune-related gene sets, whereas genes involved in cardiac contraction were downregulated. In contrast, 0.2%AHFD induced body weight loss, severe kidney dysfunction, and cardiac atrophy without functional impairment in 129×1/Sv mice. Of note, the C57BL/6 N mice exhibited metabolic abnormality and cardiac hypertrophy with diastolic dysfunction after 0.15%AHFD feeding despite mild renal dysfunction. We report a novel mouse model of CKM syndrome with dietary intervention, which exhibits fibrosis and myocardial hypertrophy in the heart. This model could be a valuable tool for analyzing the mechanism of CKM syndrome and assessing therapeutic options.

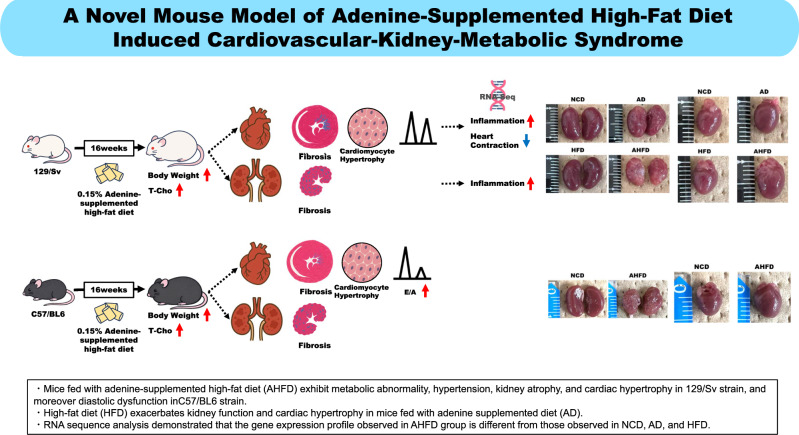

## Introduction

Cardiovascular-kidney-metabolic syndrome (CKM) is the new concept proposed by American Heart Association, which reflects multidirectional relationships among metabolic risk factors, chronic kidney disease, and the cardiovascular system [1]. High prevalence of CKM syndrome is reported in the U.S. population [2]. Although multiorgan interaction is gaining attention in the field of chronic inflammatory diseases, it was limited to interrelationships between either heart and kidney, heart and metabolic syndrome, or metabolic syndrome and kidney [3]. Different from coronary artery disease, it remains undetermined the mechanism by which synergetic effect of kidney dysfunction and metabolic syndrome on the structure and function of cardiac tissues.

In the past two decades, cardio-renal syndrome has been investigated thus far [4, 5]. However, an appropriate therapeutic strategy has not yet been established due to an absence of proper animal model for cardio-renal syndrome, notably reno-cardiac syndrome [6]. Clinical examples of chronic reno-cardiac syndrome are left ventricular hypertrophy and heart failure from chronic kidney disease (CKD)-related cardiomyopathy [4]. A report indicated that CKD mice models such as adenine-rich diet, subtotal nephrectomy, and 129 Sv strains are insufficient to induce cardiac hypertrophy, fibrosis, and dysfunction, suggesting the additional hit is required to induce reno-cardiac syndrome [6].

Recently, various two-hit models are noted in basic research in cardiology. High-fat diet and N omega-Nitro-L-arginine methyl ester hydrochloride drinking induces heart failure with preserved ejection fraction in mice [7]. Also, high-fat diet and streptozotocin intraperitoneal injection induces diabetic cardiomyopathy [8]. These findings raised the possibility that high-fat diet alters a sensitivity to cardiac remodeling and dysfunction. The adenine diet-induced CKD model is characterized by tubulointerstitial injury initiated by intratubular crystal deposition, finally leading to glomerular injury [9]. Tubulointerstitial injury is considered as the final common pathway of CKD progression regardless of etiology [10]. Clinical studies have demonstrated that tubulointerstitial injury was associated with development and progression of heart failure [11–13], suggesting its potential role in the development of CKM syndrome. Here, we established the novel mice model of CKM syndrome induced by adenine-supplemented high-fat diet.

## Materials and methods

### Animals

The study was performed with 129×1/SvJmsSlc (Japan SLC, Inc., Shizuoka, Japan) and C57BL/6 N male mice aged 5–8 weeks old. All experimental procedures were performed according to the animal welfare regulations of Yamagata University School of Medicine, and the study protocol was approved by the Animal Subjects Committee of Yamagata University School of Medicine. The investigation conformed to the Guide for the Care and Use of Laboratory Animals published by the US National Institutes of Health.

### Chow

**Fig. 1:**
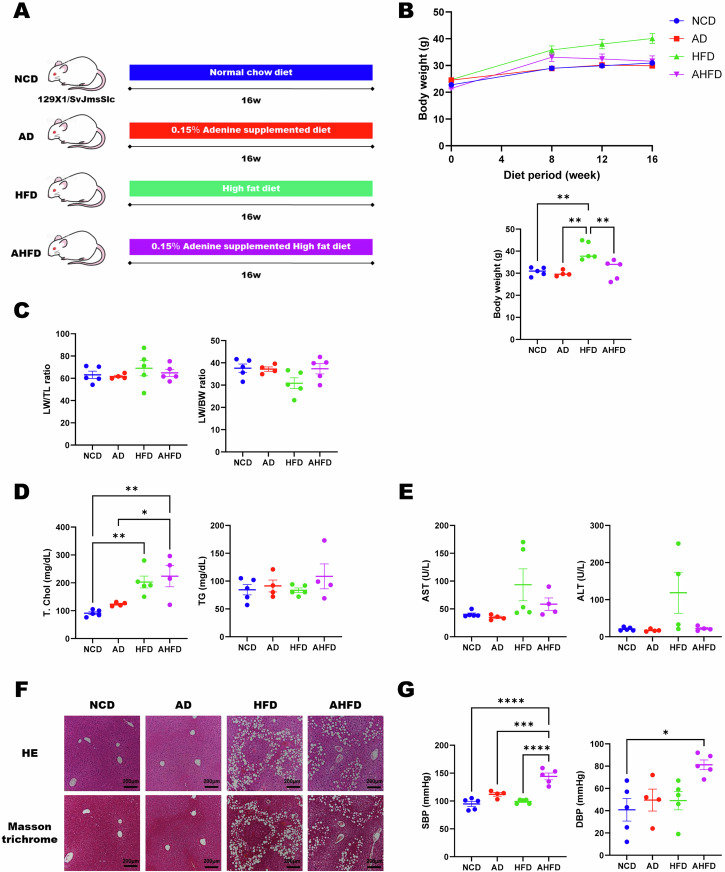
Comparisons of metabolic parameters and blood pressure among NCD, AD, HFD, and AHFD groups. **A** Study protocol. **B** Changes in body weight for 16 weeks. **C** Liver weight adjusted by tibial length or body weight at 16 weeks of diet. **D**, **E** Serum T. Chol, TG, AST, and ALT concentration at 16 weeks of diet. **F** Representative images of liver hematoxylin-eosin and Masson trichrome staining. **G** Non-invasive BP measurement at 16 weeks of diet. Results are presented as the mean ± standard error (*n* = 4–5, *P < 0.05, **P < 0.01, ***P < 0.001, ****P < 0.0001 using Tukey–Kramer test). AD, adenine-supplemented diet; AHFD, adenine-supplemented high-fat diet; ALT, alanine aminotransferase; AST, aspartate aminotransferase; SBP, systolic blood pressure; DBP, diastolic blood pressure; BW, body weight; HE, hematoxylin-eosin; HFD, high-fat diet; LW, liver weight; NCD, normal chow diet; TG, triglyceride; T. Chol, total cholesterol; TL, tibial length

Chow for adenine-supplemented diet (AD) group was 0.15% of adenine-supplemented CLEA Rodent Diet CE-2 (CLEA Japan, Inc., Tokyo, Japan), for high-fat diet (HFD) group was high fat diet 32, for adenine-supplemented high-fat diet (AHFD) group was 0.15% of adenine-supplemented high fat diet 32. CE-2 corresponded to 10% fat, 30% proteins, and 60% nitrogen free extracts. High fat diet 32 corresponded to 60% fat, 20% proteins, and 20% nitrogen free extracts. Mice were randomly assigned to normal chow diet (NCD), AD, HFD, and AHFD groups. Additional experiments using a higher adenine concentration (0.2%) combined with HFD were also performed. Total number of 129×1/Sv mice for each group was 11 in the NCD group, 5 in the AD group, 5 in the HFD group, 14 in the AHFD group, and 7 in the 0.2%AHFD group. Total number of C57BL/6 N mice for NCD and AHFD groups were 5 and 7, respectively. Each diet lasted for 16 weeks until just before the sacrifice (Fig. 1A).

### Blood pressure measurements

Systolic and diastolic blood pressure (BP) measurements were performed via non-invasive tail-cuff method using MK-2000ST (Muromachi Kikai, Tokyo, Japan). To reduce stress-related errors in the measurements, mice were trained 2 days in advance to adapt them to the experimental procedure. BP was measured at least 5 times and the middle 3 BPs were averaged. Measurements were performed within a few days before sacrifice.

### Cardiac functional measurements using echocardiography

To evaluate cardiac function, echocardiography was performed using Vevo 2100 Imaging System (FUJIFILM VisualSonics, Inc., Toronto, ON, Canada) within a few days before sacrifice. Anesthesia of mice was induced via inhalation of 3% isoflurane using an induction chamber and maintenance was facilitated via a nose mask supplying the mice with 0.5 ~ 2% isoflurane. The parasternal short axis view was used to measure left ventricular diameter, wall thickness, ejection fraction and fractional shortening. Four chamber view was used to measure mitral flow velocity.

### Blood sampling, organ harvesting, and processing

Anesthetized mice were sacrificed by puncturing inferior vena cava and removing all blood. Blood glucose was measured promptly after blood collection using NIPRO Free Style Freedom Lite (NIPRO Corp., Osaka, Japan). The collected blood was centrifuged at 3000 rpm for 15 min, the serum was transferred into fresh tube for analyses of creatinine, blood urea nitrogen, total cholesterol, triglyceride, aspartate aminotransferase, alanine aminotransferase using DRI-CHEM3500V (FUJIFILM, Tokyo, Japan). Heart, kidney and liver were excised and weighed. Apical part of the heart, lateral halves of the kidney, and one lobe of liver were frozen in liquid nitrogen and stored at −80 °C for following RNA analysis. The other parts of organs were fixed in 4% paraformaldehyde at 4 °C.

### Histological and immunofluorescence analysis

Kidney, heart and liver tissue were collected and fixed with 4% paraformaldehyde for histopathological analysis. After fixation, the samples were embedded in paraffin and sectioned into 5 µm. The kidney and heart were stained with Hematoxylin-Eosin and Masson-Trichrome. Heart sections were also stained with wheat germ agglutinin stain for the measurement of cardiomyocyte cross sectional area. Histological and immunofluorescence images were captured by biological microscope BX50 (Olympus Corp., Tokyo, Japan) or All-in-one fluorescence microscope BZ-X700 (KEYENCE Corp., Osaka, Japan). Analyzing histological staining was performed using image analyzing application BZ-X Analyzer (KEYENCE Corp., Osaka, Japan).

### Quantitative real-time PCR analysis

Approximately 20 mg of frozen pieces of the kidney and the heart were supplemented with 500 µl TRIzol reagents (15596-026, Invitrogen, MA, USA) and homogenized by tissue lyser for total RNA extraction. After phase separation achieved by chloroform, the RNA-containing aqueous phase was transferred to a new tube, precipitated by isopropanol and washed with 70% ethanol. Purified total RNA was then eluted in nuclease-free water. RNA concentration was measured using Multiscan Sky (Thermo Fisher Scientific, MA, USA), and the RNA was transcribed into cDNA with iScript Reverse Transcription Supermix for RT-qPCR (1708841, Bio-Rad Laboratories, Inc.) according to the manufacturer’s instruction. qPCRs were performed by SYBR Green protocol using SYBR Green Supermix (1725270, Bio-Rad Laboratories, Inc.) and data quantitation was performed using the ΔΔCt method. Sequences of real-time primers were described in Supplementary Table 1.

### RNA sequencing analysis

Total RNA was extracted from heart and kidney tissues using TRIzol as previously described [14]. Paired-end reads were conducted using trim_galore (v0.6.6.) (RRID: SCR_011847). The trimmed reads were mapped onto the mouse (mm10) genome sequence assembly using HISAT2 (v2.2.1) with default parameters (RRID: SCR_015530) [15]. The mapped reads were quantified using feature Counts (v2.0.1) (RRID: SCR_012919) [16]. The data have been deposited with links to BioProject accession number PRJDB35403 in the DDBJ BioProject database. All statistical analyses regarding RNA sequence were performed by iDEP 2.0 [17].

### Statistics

Data were presented as mean ± standard deviation. Statistical analysis was performed in GraphPad Prism version 9.2.0 (GraphPad Software) by applying Student’s *t*-test or ANOVA with Tukey’s multiple comparisons test. *P* < 0.05 was considered statistically significant.

## Results

### Metabolic abnormalities in 129×1/Sv mice fed with AHFD

Kaplan–Meier survival curve demonstrated a progressive decline in survival beginning after 16 weeks of AHFD intervention (Supplementary Fig. 1). Consistently, one of five mice in the AD group and two of fourteen mice in the AHFD group died before the 16-week endpoint, whereas no mortality was observed in the NCD or HFD groups.

Food intake was comparable among four groups throughout the feeding period. Average daily food consumption was approximately 3–4 g/day in the NCD and AD groups and 3–3.5 g/day in the HFD and AHFD groups similar to the previous report [18]. The body weight in the HFD group consistently increased until week 16. On the other hand, the AHFD group had a larger body weight gain than the NCD group and AD group until week 8. Later, body weight in the AHFD group tended to decrease despite no apparent reduction in food intake (Fig. 1B). As a result, body weight in the AHFD group was similar to that in the NCD and AD groups at week 16. There were no significant differences in liver weight to tibial length ratio, liver weight to body weight ratio, serum levels of triglyceride, aspartate aminotransferase, and alanine aminotransferase among four groups. However, serum total cholesterol was significantly higher in the HFD group and AHFD group compared to the remaining two groups, indicating the abnormal lipid metabolism in these groups (Fig. 1C–E). Histological analysis of the liver revealed lipid deposit and ballooning hepatocyte in the HFD and AHFD groups. No apparent hepatic fibrosis was observed in either group (Fig. 1F). Systolic BP in the AHFD group was the highest among 4 groups at 16 weeks (Fig. 1G).

### Kidney atrophy and dysfunction in 129×1/Sv mice fed with AHFD

**Fig. 2:**
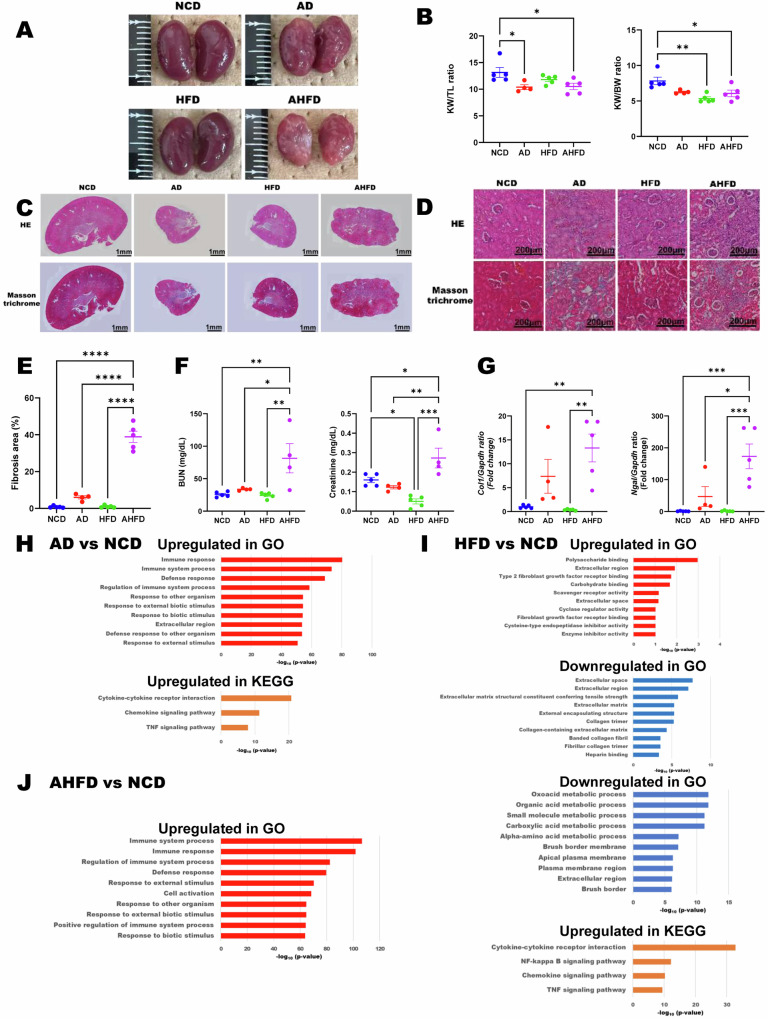
Comparisons of kidney atrophy and function among NCD, AD, HFD, and AHFD groups and RNA sequence analysis of kidney tissues. **A** Representative images of macroscopic view. **B** Kidney weight adjusted by tibial length or body weight at 16 weeks of diet. **C**, **D** Representative images of kidney hematoxylin-eosin and Masson trichrome staining. **E** Quantification of kidney fibrosis area. **F** Serum BUN and creatinine concentration at 16 weeks of diet. **G** Quantitative PCR of markers of fibrosis (*Col1*) and tubular damage (*Ngal*) normalized to *Gapdh* on kidney tissue. **H** Gene ontology analysis and KEGG pathway analysis of AD group vs. NCD group (*n* = 3 for each group). **I** Gene ontology analysis of HFD group vs. NCD group (*n* = 3 for each group). **J** Gene ontology analysis and KEGG pathway analysis of AHFD group vs. NCD group (*n* = 3 for each group). Results are presented as the mean ± standard error (*n* = 4–5, *P < 0.05, **P < 0.01, ***P < 0.001, ****P < 0.0001 using Tukey–Kramer test). AD, adenine-supplemented diet; AHFD, adenine-supplemented high-fat diet; BW, body weight; BUN, blood urea nitrogen; Col1, collagen type 1; Gapdh, Glyceraldehyde-3-phosphate dehydrogenase; HE, hematoxylin-eosin; HFD, high-fat diet; LW, liver weight; KEGG, Kyoto Encyclopedia of Genes and Genomes; KW, kidney weight; NCD, normal chow diet; Ngal, neutrophil gelatinase-associated lipocalin; TL, tibial length

Kidneys in the AHFD group were visibly atrophied and showed irregular surface and partially whitish. Similar changes were observed in the AD group, although its extent was mild (Fig. 2A). In the HFD group, the kidney weight to tibial length (KW/TL) ratio was similar to the NCD group, however, the kidney weight to body weight ratio was lower than the NCD group. KW/TL ratio was significantly lower in the AD group compared to the NCD group. The AHFD group showed the lower KW/TL ratio and kidney weight to body weight ratio than the NCD group (Fig. 2B). Histological evaluation of the kidney showed inflammatory cell infiltration, atrophy and fibrosis in the AD group and AHFD group. Of note, these findings are more severe in the AHFD group than in the AD group. Quantitative evaluation revealed a significant increase in the fibrosis area in the AHFD group (Fig. 2C–E). Blood urea nitrogen and serum creatinine were the highest in the AHFD group among four groups (Fig. 2F). Kidney mRNA expression of fibrosis marker (*Col1*) and tubular injury marker (*Ngal*) were significantly increased in the AHFD group (Fig. 2G).

### RNA sequence analysis of the Kidney

RNA-sequence analysis was performed on kidney tissues of each group. Principal component analysis and volcano plots in the AD, HFD, and AHFD group compared to the NCD group were shown in Supplementary Fig. 2. GO analysis comparing the AD and NCD group indicated that the upregulated differentially expressed genes (DEGs) were associated with GO terms related to immune response. Cytokine-cytokine receptor interaction, chemokine signaling pathway, and tumor necrosis factor (TNF) signaling pathway were detected as the upregulated pathways in the Kyoto Encyclopedia of Genes and Genomes (KEGG) pathway analysis in the AD compared to the NCD group (Fig. 2H). GO analysis comparing the HFD and NCD group indicated that the downregulated DEGs were associated with GO terms related to the extracellular matrix (Fig. 2I). GO analysis comparing the AHFD and NCD group showed the upregulated DEGs were associated with GO terms related to immune response, while the downregulated DEGs were associated with GO terms related to metabolism (Fig. 2J). Cytokine-cytokine receptor interaction, NF-κB signaling pathway, chemokine signaling pathway, and TNF signaling pathway were detected as the upregulated pathways in the AHFD compared to the NCD group (Fig. 2J).

### Cardiac hypertrophy and fibrosis in 129×1/Sv mice fed with AHFD

**Fig. 3:**
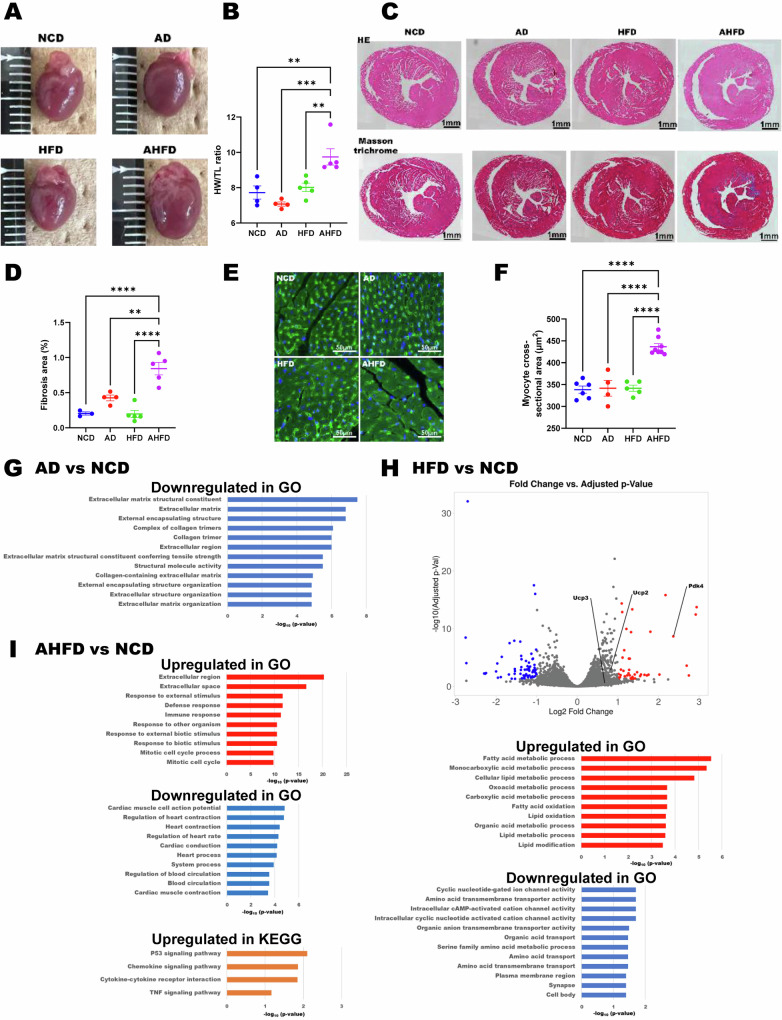
Comparison of cardiac hypertrophy among NCD, AD, HFD, and AHFD groups and RNA sequence analysis of heart tissues. **A** Representative images of macroscopic view. **B** Heart weight adjusted by tibial length at 16 weeks of diet. **C** Representative images of hematoxylin-eosin and Masson trichrome staining (scale bar = 1 mm). **D** Quantification of fibrosis area. **E** Representative images of immunofluorescence staining for wheat germ agglutinin and DAPI (scale bar = 50 µm). **F** Quantification of myocyte cross-sectional area. **G** Gene ontology analysis of AD group vs. NCD group (*n* = 3 for each group). **H** Volcano plot and gene ontology analysis of HFD group vs. NCD group (*n* = 3 for each group). **I** Gene ontology analysis and KEGG pathway analysis of AHFD group vs. NCD group (*n* = 3 for each group). Results are presented as the mean ± standard error (*n* = 3–5, *P < 0.05, **P < 0.01, ***P < 0.001, ****P < 0.0001 using Tukey–Kramer test). AD, adenine-supplemented diet; AHFD, adenine-supplemented high-fat diet; HE, hematoxylin-eosin; HFD, high-fat diet; HW, heart weight; KEGG, Kyoto Encyclopedia of Genes and Genomes; NCD, normal chow diet; TL, tibial length

The length between base and apex in the heart from the AHFD group tended to be visually long (Fig. 3A). Heart weight to tibial length ratio was significantly increased in the AHFD group than other groups (Fig. 3B). In histological analysis, fibrosis was observed in the AHFD group hearts not only perivascular areas but also interstitial areas, nevertheless little interstitial fibrosis was observed in other groups (Fig. 3C). This was also demonstrated by the fact that the fibrosis area was significantly increased in the AHFD group than the other groups (Fig. 3D). Microscopic analysis revealed that the cross-sectional area of cardiomyocytes increased considerably in the AHFD group than in the other groups (Fig. 3E–F). Unexpectedly, there were no significant differences in the echocardiographic parameters among four groups (Supplementary Table 2).

### RNA sequence analysis of the Heart

RNA sequence analysis was performed on cardiac tissues of each group. Principal component analysis and volcano plots in the AD, HFD, and AHFD group compared to the NCD group were shown in Fig. 3H and Supplementary Fig. 3. GO analysis comparing between the AD and NCD group showed that downregulated DEGs were associated with GO terms related to the extracellular matrix (Fig. 3G). GO analysis comparing the HFD and NCD group indicated that the upregulated DEGs were associated with GO terms related to lipid metabolism (Fig. 3H). GO analysis comparing the AHFD and NCD group indicated that the upregulated DEGs were associated with GO terms related to the extracellular matrix and immune response, and downregulated DEGs were associated with GO terms related to the cardiac contraction (Fig. 3I). The p53 pathway, chemokine signaling pathway, cytokine-cytokine receptor interaction, and TNF signaling pathway were detected as the upregulated pathways in the KEGG pathway analysis in the AHFD compared to the NCD group (Fig. 3I). *Pyruvate dehydrogenase kinase isozyme 4* (*Pdk4*) was identified as the common DEG in the HFD and AHFD groups.

### Differences in RNA expression between the AHFD and AD groups

**Fig. 4:**
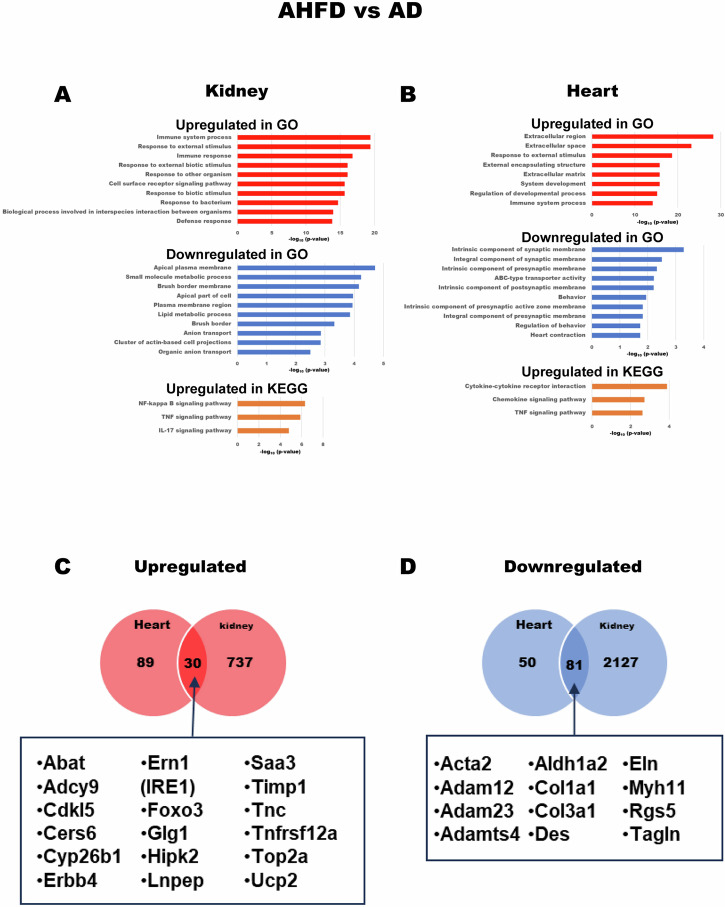
RNA sequence analysis between AHFD and AD group. **A** Gene ontology analysis and KEGG pathway analysis of AHFD group vs. AD group of the kidney. **B** Gene ontology analysis and KEGG pathway analysis of AHFD group vs. AD group of the heart. (*n* = 3 for each group) Overlap of differentially expressed genes between heart and kidney. Venn diagram of upregulated (**C**) and downregulated (**D**) genes in the heart and kidney. Representative common upregulated and downregulated genes were shown. AD, adenine-supplemented diet; AHFD, adenine-supplemented high-fat diet; KEGG, Kyoto Encyclopedia of Genes and Genomes

To examine the effect of a high-fat diet on the susceptibility to kidney and cardiac remodeling, we compared the RNA expression between AHFD and AD groups. Interestingly, the immune system process and response to external stimulus in GO analysis and TNFα signaling pathway in KEGG analysis were common upregulated gene sets in both heart and kidney (Fig. 4A–B).

### Common differentially expressed genes in the heart and kidney

Comparative RNA-seq analysis between the heart and kidney tissues of the AHFD group identified 30 commonly upregulated genes (Fig. 4C). These included genes related to endoplasmic reticulum stress (*Ern1*), mitochondrial and metabolic regulation (*Ucp2, Abat*), and inflammation and tissue remodeling (*Saa3, Timp1, Tnc*), suggesting the presence of shared stress-response pathways underlying cardiovascular-kidney-metabolic interactions. In contrast, 81 genes were commonly downregulated in both organs (Fig. 4D), including those associated with smooth muscle and vascular structure (*Acta2, Myh11, Tagln*) and extracellular matrix components (*Col1a1, Col3a1, Eln*), indicating disruption of structural remodeling processes.

### Effect of 0.2%AHFD on cardiac atrophy in 129×1/Sv mice

**Fig. 5:**
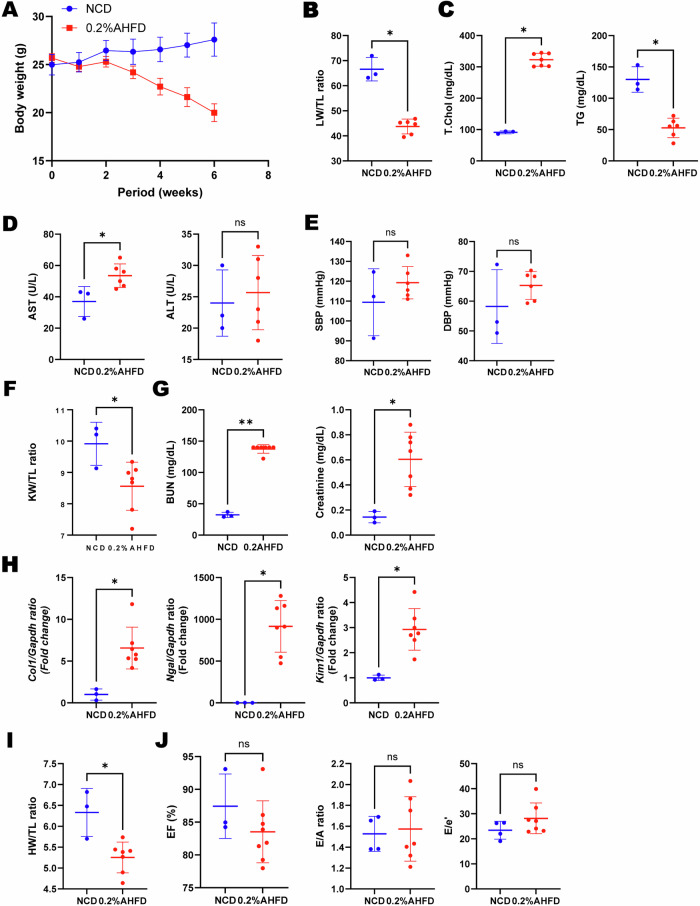
Comparisons of metabolic, renal and cardiac parameters at 6 weeks of diet between NCD and 0.2%AHFD groups. **A** Changes in body weight for 6 weeks. **B** Liver weight adjusted by tibial length. **C**, **D** Serum T. Chol, TG, AST and ALT concentration. **E** Non-invasive BP measurement. **F** Kidney weight adjusted by tibial length. **G** Serum BUN and creatinine. **H** Quantitative PCR of markers of fibrosis (*Col1*) and tubular damage (*Ngal, Kim1*) normalized to *Gapdh* on kidney tissue. **I** Heart weight adjusted by tibial length. **J** Echocardiographic parameters. Results are presented as the mean ± standard error (*n* = 3–7, *P < 0.05, **P < 0.01, ***P < 0.001, ****P < 0.0001 using Student’s *t*-test). AHFD, adenine-supplemented high-fat diet; ALT, alanine aminotransferase; AST, aspartate aminotransferase; SBP, systolic blood pressure; DBP, diastolic blood pressure; BUN, blood urea nitrogen; Col1, collagen type 1; EF, ejection fraction; E/A, the ratio of the early transmitral flow velocity and late transmitral flow velocity; E/e’, early mitral inflow velocity and peak velocity of early diastolic mitral annulus motion by tissue Doppler; HE, hematoxylin-eosin; HW, heart weight; Kim1, Kidney injury molecule 1; KW, kidney weight; LW, liver weight; NCD, normal chow diet; Ngal, neutrophil gelatinase-associated lipocalin; TG, triglyceride; T. Chol, total cholesterol; TL, tibial length

To determine an appropriate adenine concentration for modeling CKM syndrome, we also tested 0.2%AHFD in 129×1/Sv mice. Different from the 0.15%AHFD, mice receiving 0.2%AHFD exhibited progressive body weight loss throughout the experimental period (Fig. 5A).

Liver weight to tibial length ratio in the 0.2%AHFD group was significantly reduced (Fig. 5B), while total cholesterol and aspartate aminotransferase levels were increased (Fig. 5C–D). BP was similar between the 0.2%AHFD and NCD groups (Fig. 5E). KW/TL ratio was significantly reduced in the 0.2%AHFD group (Fig. 5F). Blood urea nitrogen and serum creatinine levels were markedly elevated in 0.2%AHFD group (Fig. 5G). Consistently, kidney mRNA expression of fibrosis (*Col1*) and tubular injury marker (*Ngal* and *Kim1*) were significantly upregulated in the 0.2%AHFD group (Fig. 5H). Heart weight to tibial length was significantly reduced in the 0.2%AHFD group (Fig. 5I). However, cardiac function was not altered (Fig. 5J). These findings indicated that 0.2%AHFD induces cardiac atrophy together with severe weight loss and kidney dysfunction.

### Cardiac hypertrophy and diastolic dysfunction in C57BL/6 N mice fed with 0.15%AHFD

**Fig. 6:**
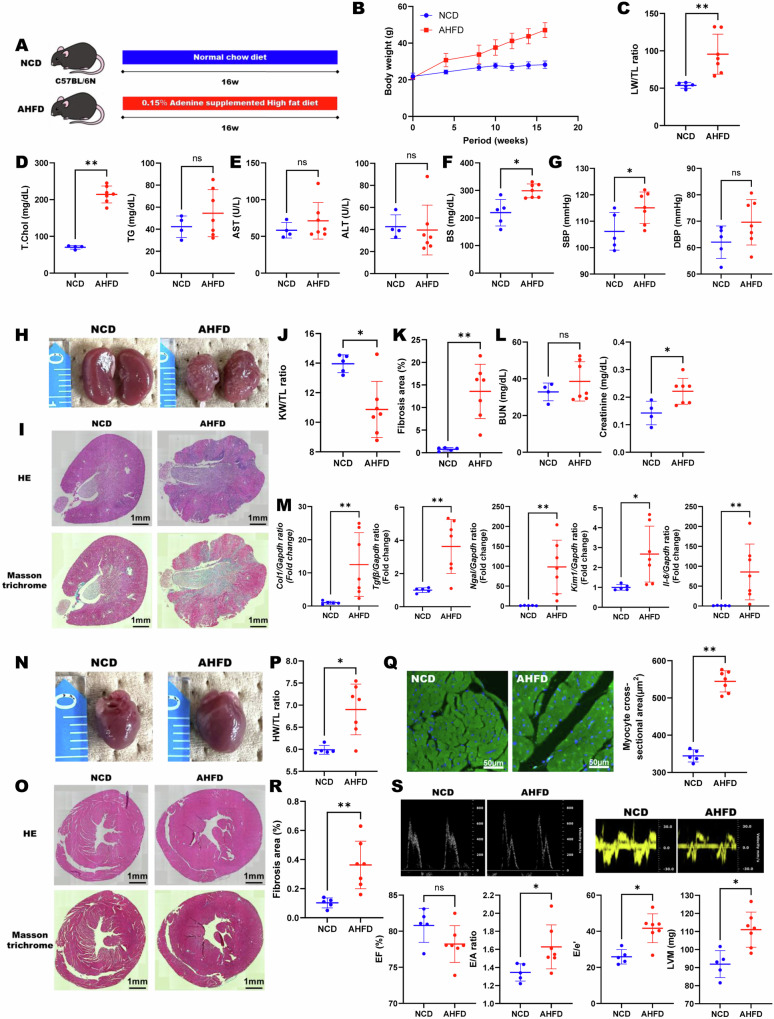
Comparisons of metabolic, renal and cardiac parameters at 16 weeks of diet between NCD and 0.15%AHFD groups in C57BL/6 N mice. **A** Study protocol. **B** Changes in body weight for 16 weeks. **C** Liver weight adjusted by tibial length. **D**–**F** Serum T. Chol, TG, AST, ALT and BS concentration. **G** Non-invasive BP measurement. **H** Representative images of kidney macroscopic view. **I** Representative images of kidney hematoxylin-eosin and Masson trichrome staining. **J** Kidney weight adjusted by tibial length. **K** Quantification of kidney fibrosis area. **L** Serum BUN and creatinine concentration. **M** Quantitative PCR of markers of fibrosis (*Col1, Tgfβ*), tubular damage (*Ngal, Kim1*) and inflammation (*Il-6*) normalized to *Gapdh* on kidney tissue. **N** Representative images of heart macroscopic view. **O** Representative images of heart hematoxylin-eosin and Masson trichrome staining. **P** Heart weight adjusted by tibial length at 16 weeks of diet. **Q** Representative images of immunofluorescence staining for wheat germ agglutinin and DAPI (scale bar = 50 µm). Quantification of myocyte cross-sectional area. **R** Quantification of heart fibrosis area. **S** Representative echocardiographic image and echocardiographic parameters. Results are presented as the mean ± standard error (*n* = 5–7, *P < 0.05, **P < 0.01, ***P < 0.001, ****P < 0.0001 using Student’s t-test). AHFD, adenine-supplemented high-fat diet; ALT, alanine aminotransferase; AST, aspartate aminotransferase; SBP, systolic blood pressure; DBP, diastolic blood pressure; BS, Blood sugar; BUN, blood urea nitrogen; Col1, collagen type 1; EF, ejection fraction; E/A, the ratio of the early transmitral flow velocity and late transmitral flow velocity; E/e’, early mitral inflow velocity and peak velocity of early diastolic mitral annulus motion by tissue Doppler; Gapdh, Glyceraldehyde-3-phosphate dehydrogenase; HE, hematoxylin-eosin; HW, heart weight; Il-6, interleukin-6; Kim1, Kidney injury molecule 1; KW, kidney weight; LVM, left ventricular mass; LW, liver weight; NCD, normal chow diet; Ngal, neutrophil gelatinase-associated lipocalin; TG, triglyceride; T. Chol, total cholesterol; Tgfβ, transforming growth factor β; TL, tibial length

In C57BL/6 N mice, the AHFD group exhibited a significant increase in body weight compared with the NCD group (Fig. 6B). Furthermore, C57BL/6 N mice demonstrated sustained weight gain up to week 16. The liver weight to tibial length ratio was significantly increased in the AHFD group (Fig. 6C). Serum total cholesterol and fasting blood glucose levels were significantly elevated in the AHFD group (Fig. 6D–F). The systolic BP was significantly elevated by approximately 10 mmHg in the AHFD group (Fig. 6G).

Macro- and microscopically, kidneys showed marked atrophy in the AHFD group. The KW/TL ratio was significantly decreased in the AHFD group (Fig. 6H–J). Histological analysis revealed increased fibrosis area in the AHFD group (Fig. 6I and K). Serum creatinine levels were significantly increased in the AHFD group, but not blood urea nitrogen (Fig. 6L). Kidney RNA expression of fibrosis (*Col1a1, Tgfβ*), tubular injury (*Ngal, Kim1*), and inflammatory (*Il6*) markers were upregulated in the AHFD group (Fig. 6M).

Macro- and microscopical findings showed cardiac hypertrophy and fibrosis in the AHFD group. Heart weight to tibial length ratio was significantly increased in the AHFD group (Fig. 6N–P). Cardiomyocyte cross-sectional area was significantly larger in the AHFD than in NCD group (Fig. 6Q). Histological analysis demonstrated increased fibrosis area in the AHFD group (Fig. 6O and R). Importantly, echocardiogram showed the increased E/A and E/e′ ratios in AHFD group compared to the NCD group despite preserved ejection fraction (Fig. 6S). These findings indicated that AHFD induced cardiac hypertrophy with diastolic dysfunction in C57BL/6 N mice.

## Discussion

The novel and important findings of this study are (1) that mice fed with AHFD exhibit metabolic abnormality, hypertension, kidney atrophy, and cardiac hypertrophy; (2) HFD exacerbates kidney function and cardiac hypertrophy in 129×1/Sv mice fed with AD; (3) RNA sequence analysis demonstrated that the gene expression profile observed in AHFD group is different from those observed in NCD, AD, and HFD groups; (4) 0.2%AHFD induces weight loss, severe kidney dysfunction, and cardiac atrophy in 129×1/Sv mice; and (5) AHFD induces metabolic abnormality, mild kidney dysfunction, and cardiac hypertrophy with diastolic dysfunction in C57BL/6 N mice. Numerous studies have been conducted on model animals for reno-cardiac syndrome and cardiometabolic syndrome to examine its effects on the cardiac remodeling and function using various established CKD or diabetes models [19–22]. We developed a new CKM stage 3 mice models by feeding AHFD to 129×1/Sv and C57BL/6 N mice.

### The effect of AD on the kidney and heart

Adenine-rich diet reportedly induces tubulointerstitial nephropathy via TNFα signaling [9, 23]. RNA-seq analysis of kidney tissues showed upregulation of TNFα signaling in the present study. Thus, AD and AHFD groups may exhibit similar atrophy and fibrosis patterns via TNFα signaling.

A report indicated that 20-week 0.15% adenine-rich diet administration induces cardiac hypertrophy, fibrosis, and 7% reduction in ejection fraction in C57BL/6 N mice [24]. Wollenhaupt et al. examined the effects of CKD on cardiac remodeling and function using subtotal nephrectomy and several concentrations of adenine-rich diet [6]. However, cardiac remodeling and function were not observed in those models, similar to the present study. Therefore, it is still controversial whether an adenine-rich diet is a suitable model for reno-cardiac syndrome. In the light of gene expression, the gene sets related to oxidative stress, inflammation, extracellular matrix, collagen/fibrosis, and mitochondrial function were reportedly upregulated (Supplementary Fig. S3). We reconfirmed these findings in the heart from mice fed with AD. Despite changes in gene sets were observed, it was suggested that the additional hits are required to induce obvious cardiac remodeling in mice CKD model.

### The effect of HFD on the kidney and heart

Body weight and total cholesterol levels were significantly increased in the HFD group compared to NCD group, suggesting the metabolic syndrome was successfully induced by HFD in the present study. Roberts et al. reported that the mice fed with HFD exhibit the significant increase in heart weight and cardiomyocyte size without cardiac dysfunction [25]. Calligaris et al. indicated that mice fed with 16 months HFD did not show any cardiac pathology by echocardiography [26]. These reports suggested that HFD is not sufficient to induce cardiac dysfunction, while cardiac remodeling is inducible. Contrary with previous reports, we did not show an increase in heart weight or cardiac hypertrophy with only HFD group.

Previous studies have suggested that insulin resistance and mitochondrial impairment of metabolic regulation are involved in cardiac hypertrophy and decreased cardiac function. In the light of gene expression, it was reported that *Pdk4*, which is a kinase of pyruvate dehydrogenase with multifactorial function in mitochondrial metabolism predominantly expressed in heart muscle [27], was overexpressed in obese mice [26]. Similarly, the expression of *Pdk4* was increased in the hearts from mice fed with HFD by RNA sequence analysis in the present study.

### HFD augmented susceptibility to AD-induced cardiac remodeling

We found that not only kidney dysfunction but also cardiac hypertrophy was pronounced in the AHFD group compared to AD group in 129×1/Sv mice. Elevations of BP and total cholesterol in AHFD supported the notion that metabolic abnormalities augmented kidney dysfunction and cardiac remodeling. This is also indicated by the increase in the RNA expression of inflammatory cytokine and chemokines in both kidney and heart, which are involved in the pathogenesis of heart failure [28].

### AHFD and CKM syndrome

Recent report showed that 0.2%AHFD induces CKM phenotype characterized by metabolic abnormality, kidney dysfunction and peripheral artery disease in C57BL/6 N mice. Although cardiac fibrosis was observed, the data on cardiac hypertrophy and function were not provided [29]. Unfortunately, cardiac and metabolic phenotypes of 129×1/Sv mice fed with 0.2%AHFD observed in this study were clearly different from those in human CKM syndrome. Thus, we utilized 0.15%AHFD in this study.

Carvalho et al. indicated a multiple hit model of CKM syndrome by combining unilateral nephrectomy and high-salt-sugar-fat diet for 12 or 20 weeks in mice [30]. Their model shows about 15 mmHg BP elevation, moderate kidney dysfunction, 1.1 folds increase in cardiomyocyte cross-sectional area, cardiac perivascular fibrosis, and mild systolic dysfunction at 20 weeks. We showed more pronounced BP elevation, myocardial hypertrophy, and interstitial fibrosis in 129X/Sv mice fed with 0.15%AHFD. Unexpectedly, cardiac function was preserved despite the down-regulation of gene sets involved in heart contraction. Importantly, despite only modest systolic BP elevation, the phenotypic characteristics in C57BL/6 N mice fed with 0.15%AHFD were highly consistent with the pathophysiological hallmarks of heart failure with preserved ejection fraction. Heart failure with preserved ejection fraction is increasingly recognized as a systemic disorder driven by metabolic stress, low-grade inflammation, and multiorgan dysfunction rather than pressure overload alone [31]. Our results raised the possibility that metabolic disorder and renal tubular injury induced by AHFD feeding act as upstream drivers of cardiac remodeling and diastolic dysfunction. The observation that cardiac dysfunction emerged in the absence of severe hypertension supports the concept that non-hemodynamic mechanisms play a central role in the development of CKM syndrome. Collectively, 0.15%AHFD induced CKM model is equivalent to CKM stage 3: subclinical heart failure [1].

Cardiovascular, kidney, and metabolic diseases interact with each other at the pathophysiological level, leading to a clinical overlap [32]. Accumulating evidence indicated the inflammation plays a key role in these interactions [3, 5, 33–36]. We showed upregulation of inflammation-related genes in both the heart and kidneys in the AHFD group. Thus, the synergistic effect of AD and HFD on the development of mice CKM syndrome is in part explained by inflammation. Further research exploring a wide range of CKM syndrome models is required to clarify its pathology.

## Conclusion

This study provides a new CKM syndrome model option. This double-hit model requires only dietary load and shows features of CKM syndrome. This model could be valuable for analyzing the mechanism and developing therapeutic options of CKM syndrome.

## Supplementary information

Supplementary information
