# Supplementary material for: A novel mouse model of adenine-supplemented high-fat diet induced cardiovascular-kidney-metabolic syndrome
Source: Hypertens Res. 2026 Apr 27;49(7):2201–14. doi: 10.1038/s41440-026-02645-1 (PMC13333507; doi:10.1038/s41440-026-02645-1)
Supplement: Supplementary file 1 — Supplementary information [file 41440_2026_2645_MOESM1_ESM.pdf]

A Novel Mouse Model of Adenine-Supplemented High-Fat Diet Induced Cardiovascular-Kidney-Metabolic Syndrome

Hiroe Ono, MD[a], Yoichiro Otaki, MD, PhD,[a], Tetsu Watanabe, MD, PhD,[a], Ryuhei Yamaguchi, MD[a], Tomohiro Takehara, MD[a], Shingo Tachibana, MD[a], Jun Goto, MD, PhD[a], Takanori Arimoto, MD, PhD[a], Haruki Ochi, PhD[b], and Masafumi Watanabe, MD, PhD[a]

[a]Department of Cardiology, Pulmonology, and Nephrology; Yamagata University School of Medicine; Yamagata, Japan; [b]Graduate School of Science and Faculty of Science, Kobe University, Hyogo, Japan

Short title: Adenine-HFD induced CKM model

Correspondence: Yoichiro Otaki, MD, PhD

Department of Cardiology, Pulmonology, and Nephrology, Yamagata University School of Medicine, 2-2-2 Iida-Nishi, Yamagata, Japan 990-9585

Phone: +81-23-628-5302; Fax: +81-23-628-5305; E-mail: y-otaki@med.id.yamagata-u.ac.jp

Word count of the manuscript: 5,000 words, **Figure:** 6, **Supporting information:** 1

Category of the manuscript: Basic science

Supplementary tables.

Supplementary table 1. Sequences of real-time PCR primers

Gene	Gene Abbreviation	Forward primer	Reverse primer
Gapdh	Glyceraldehyde-3-phosphate dehydrogenase	TAT GAT GAC ATC AAG AAG GT	AAG AGT GGG AGT TGC TGT TG
Col1	collagen 1	AGC ACG TCT GGT TTG GAG AG	GAC ATT AGG CGC AGG AAG GT
Ngal	neutrophil gelatinase-associated lipocalin	ATG TCA CCT CCA TCC TGG TCA G	GCC ACT TGC ACA TTG TAG CTC TG
Kim1	Kidney injury molecule 1	CTG GAA TGG CAC TGT GAC ATC C	GCA GAT GCC AAC ATA GAA GCC C
Il-6	Interleukin-6	TAC CAC TTC ACA AGT CGG AGG C	CTG CAA GTG CAT CAT CGT TGT TC
Tgfβ	Transforming growth factor β	CAC AAG AGC AGT GAG CGC TGA A	TGA TAC GCC TGA GTG GCT GTC T

Supplementary table 2. Comparisons of echocardiographic parameters at 16 weeks of diet among NCD, AD, HFD, and AHFD groups.

	NCD	AD	HFD	AHFD
EF (%)	70.0±1.2	69.6±0.8	68.8±1.2	69.6±1.8
FS (%)	38.6±0.4	38.4±0.6	37.6±0.5	38.1±0.4
E/A	1.36±0.12	1.33±0.16	1.45±0.15	1.56±0.10
E/e’	25.3±2.4	32.4±2.9	29.0±3.9	26.5±5.2

AD, adenine-supplemented diet; AHFD, adenine-supplemented high-fat diet; EF, ejection fraction; FS, fractional shortening; E/A, the ratio of the early transmitral flow velocity and late transmitral flow velocity; E/e’, early mitral inflow velocity and peak velocity of early diastolic mitral annulus motion by tissue Doppler; HFD, high-fat diet; NCD, normal chow diet.

Supplementary figures and results

Supplementary figure 1. Kaplan-Meier survival curve of 129X1/Sv mice fed 0.15% AHFD.

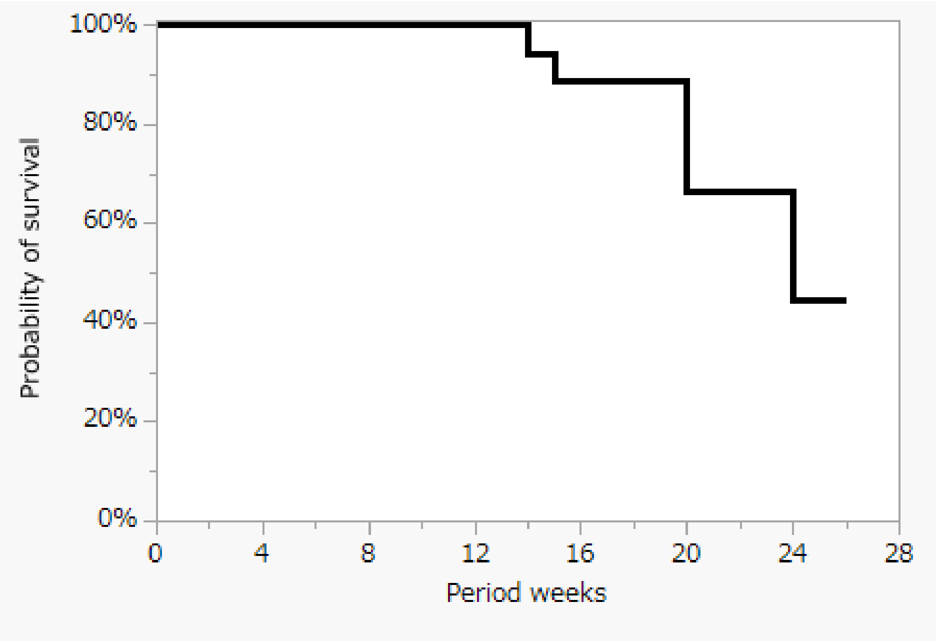

All deceased mice had low weight gain from the early phase and at the time of death, one kidney was markedly atrophied, so there was a marked left-right difference in the size of the kidneys. These findings suggest that ischemia or hydronephrosis in a single kidney may have led to a rapid decline in renal function, ultimately resulting in death. Although previous studies using adenine diets have not reported similar findings, the fact that this phenomenon was observed not only in the AHFD group but also in the AD group in the present study, suggesting that it is likely attributable to the effects of adenine.

Supplementary figure 2. RNA sequence analysis of kidney tissues collected from NCD, AD, HFD, and AHFD.

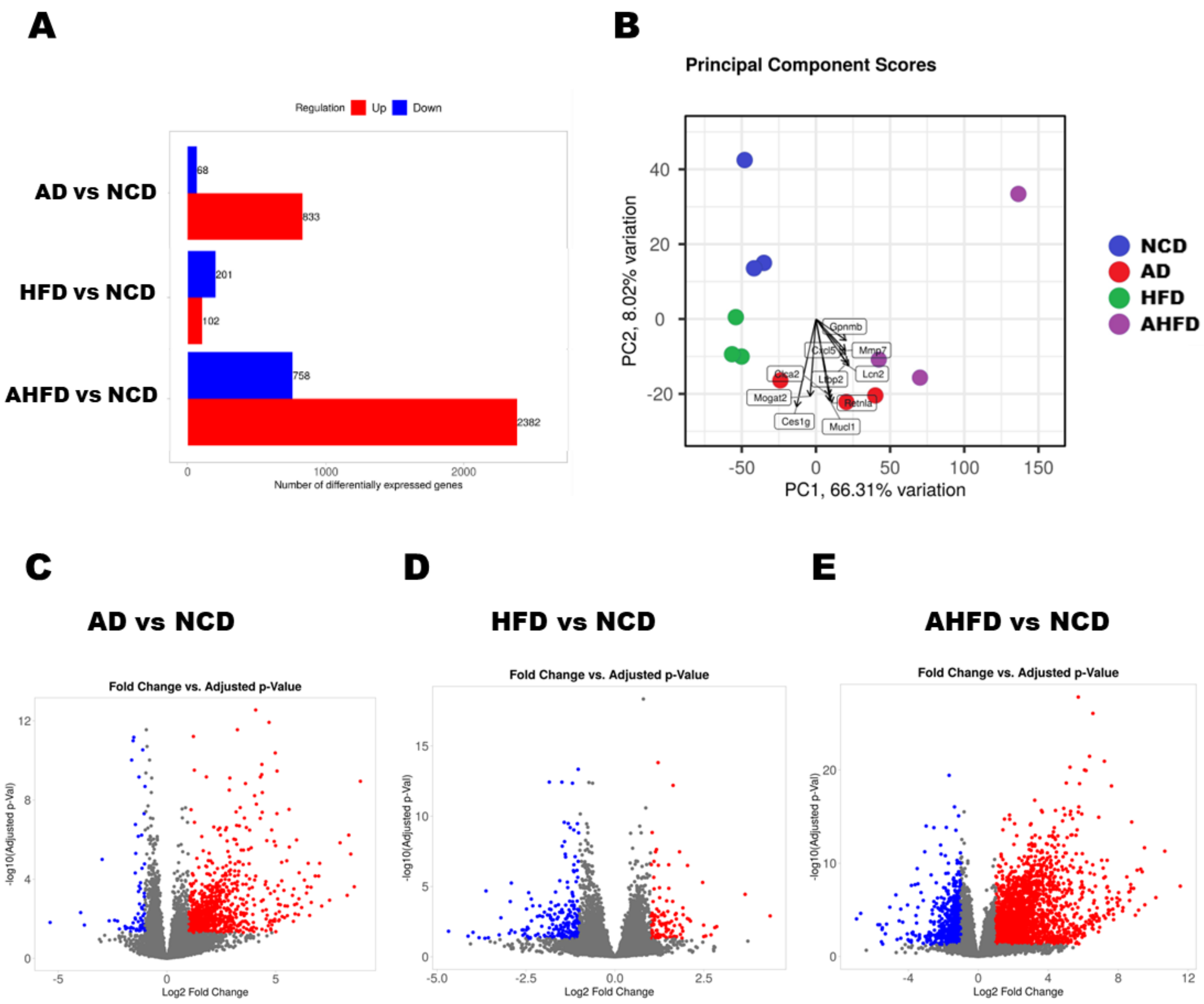

(A) Number of differentially expressed genes ($\log_2$ (fold change) > 1 or < -1; adjusted p-value < 0.05). **(B)** Principal component analysis. According to the pathway analysis of PCA rotation, PC1 indicates cell activation, immune system process, and immune response, and PC2 indicates organic acid metabolic process and carboxylic acid metabolic process. Each point represents a sample. Each color represents each group; the blue points are NCD group, the red points are AD group, the green points

are HFD group, the purple points are AHFD group. Labeled genes are that affect the principal components, and arrows are represent the direction and degree of their influence. **(C)** Volcano plot of AD group vs. NCD group. **(D)** Volcano plot of HFD group vs. NCD group. **(E)** Volcano plot of AHFD group vs. NCD group. AD, adenine-supplemented diet; AHFD, adenine-supplemented high-fat diet; HFD, high-fat diet; NCD, normal chow diet.

This revealed 833 significantly upregulated and 68 downregulated genes (AD vs. NCD group), 102 upregulated and 201 downregulated genes (HFD vs. NCD group), 2,382 upregulated and 758 downregulated genes (AHFD vs. NCD group) (Figure S2A). Principal component analysis showed that each of the four groups exhibited different gene expression (Figure S2B). Differentially expressed genes (DEGs) in the AD, HFD, and AHFD group compared to the NCD group were described in the volcano plots (Figures S2C-E).

Supplementary figure 3. RNA sequence analysis of kidney tissues collected from NCD, AD, HFD, and AHFD.

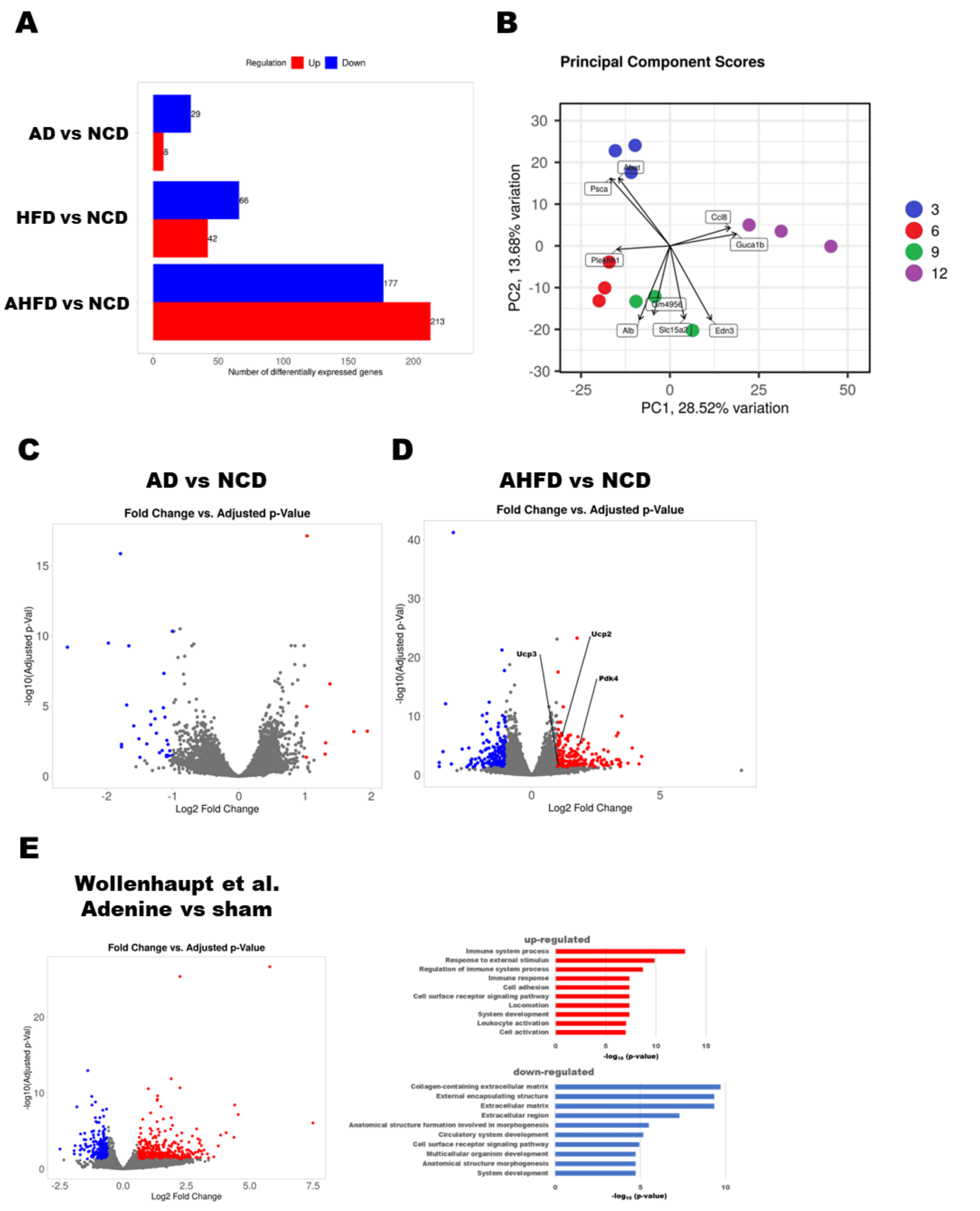

(A) Number of differentially expressed genes ($\log_2$ (fold change) > 1 or < -1; adjusted p-value < 0.05). **(B)** Principal component analysis. According to the pathway analysis of PCA rotation, PC1 indicates cell activation, immune system process, and immune response, and PC2 indicates organic acid metabolic process and carboxylic acid metabolic process. Each point represents a sample. Each color represents each group; the blue points are NCD group, the red points are AD group, the green points are HFD group, the purple points are AHFD group. Labeled genes are that affect the principal components, and arrows are represent the direction and degree of their influence. **(C)** Volcano plot of AD group vs. NCD group. **(D)** Volcano plot of AHFD group vs. NCD group. **(E)** Volcano plot and gene ontology analysis of the previously reported adenine group versus sham group from the reanalysis of public data. AD, adenine-supplemented diet; AHFD, adenine-supplemented high-fat diet; HFD, high-fat diet; NCD, normal chow diet.

This revealed 9 significantly upregulated and 29 downregulated genes (AD vs. NCD group), 42 upregulated and 66 downregulated genes (HFD vs. NCD group), 213 upregulated and 177 downregulated genes (AHFD vs. NCD group) (Figure S3A). Principal component analysis revealed that each of the four groups exhibited different phenotypes in the heart (Figure S3B). DEGs in the AD, HFD, and AHFD group compared to the NCD group were described in volcano plots (Figures 3H, S3C and D).

We reanalyzed public data regarding RNA sequence analysis of cardiac tissues from 129/Sv

mice fed with the adenine-supplemented diet for 13 weeks and compared our RNA sequence data in the AD group [1]. The AD group of present study and adenine group of previous study showed a common decrease in the expression of genes related to the extracellular matrix. Adenine group of previous study also showed an increase in the expression of genes related to immune response (Figure S3E).

Reference

1. Wollenhaupt J, Frisch J, Harlacher E, Wong DWL, Jin H, Schulte C, et al. Pro-oxidative priming but maintained cardiac function in a broad spectrum of murine models of chronic kidney disease. Redox Biol. 2022;56:102459.
